# Highlights from the Third European International Society for Computational Biology (ISCB) Student Council Symposium 2014

**DOI:** 10.1186/1471-2105-16-S3-A1

**Published:** 2015-02-13

**Authors:** Margherita Francescatto, Susanne MA Hermans, Sepideh Babaei, Esmeralda Vicedo, Alexandre Borrel, Pieter Meysman

**Affiliations:** 1 Department of Genome Biology for Neurodegenerative Diseases, German Center for Neurodegenerative Diseases (DZNE), Tübingen, Germany; 2 Computational Discovery and Design (CDD) group, Centre for Molecular and Biomolecular Informatics (CMBI), Radboudumc, Nijmegen, The Netherlands; 3 Delft Bioinformatics Lab, Delft University of Technology, The Netherlands; 4 Department for Bioinformatics and Computational Biology, Institut für Informatik, TU München, Munich, Germany; 5 INSERM, UMRS-973, MTi, Paris, France; 6 University Paris Diderot, Sorbonne Paris Cité, UMRS-973, MTi, Paris, France; 7 University of Helsinki, Division of Pharmaceutical Chemistry, Faculty of pharmacy, Finland; 8 Department of Mathematics and Computer Science, University of Antwerp, Antwerp, Belgium; 9 Biomedical Informatics Research Center Antwerp (biomina), University of Antwerp/Antwerp University Hospital, Edegem, Belgium

## Abstract

In this meeting report, we give an overview of the talks, presentations and posters presented at the third European Symposium of the International Society for Computational Biology (ISCB) Student Council. The event was organized as a satellite meeting of the 13th European Conference for Computational Biology (ECCB) and took place in Strasbourg, France on September 6^th^, 2014.

## Introduction

The ISCB Student Council (SC) is the student organization of the International Society for Computational Biology. Its members are typically PhD students in the fields of bioinformatics or computational biology, but include as well scientists in different stages of their career. They come from all around the world and share a passion for bioinformatics and computational biology. The mission of the SC is to support the development of the next generation of computational biologists. This is achieved through the provision of scientific events, networking opportunities, soft-skills training, educational resources and career advice, while attempting to influence policy processes affecting science and education.

The European Student Council Symposium (ESCS) is one of the activities organized by the SC as a satellite meeting accompanying the European Conference for Computational Biology (ECCB). It is therefore the European spin-off of the Student Council Symposium (SCS), which celebrated its 10^th ^anniversary this year [1] and is a satellite meeting of the annual Intelligent Systems for Molecular Biology (ISMB) conference. The ESCS has been organized every two years, when ECCB was not conjoined with ISMB, since 2010.

## Scope and format of the meeting

This year, the 3^rd ^ESCS took place in Strasbourg, France on September 6^th ^in conjunction with the 13th ECCB conference. The main goal of the meeting was to create opportunities for young researchers to meet and discuss with peers from all over the world, so that ideas could be exchanged and networks built. In addition three highly successful principal investigators were invited to deliver inspiring keynote talks.

We received more than 30 abstract submissions from students who wished to present their work at the symposium. These submissions were peer-reviewed by an independent program committee, and eight abstracts were selected for oral presentations. Another eighteen abstracts were selected to be presented as a poster. Thanks to the generous contributions of our sponsors, we were able to provide four travel fellowships to support student attendance to ESCS. Overall, almost 30 delegates from 13 different countries attended the symposium and the program included three inspiring keynote lectures, eight contributed student presentations, and a lively poster session. The oral presentations were divided into three themed sessions, namely Modeling, Systems Biology, and Networks and Statistics. For the first time in an event organized by the SC, the five delegates with the best posters were given the opportunity to present their work in a flash presentation. This ensured that all attendees had the chance to hear and see the top poster selection unconstrained by the population limits intrinsic to a normal poster presentation. All abstracts of the accepted oral presentations are included in this meeting report. Abstracts of the poster presentations can be found online in the symposium booklet http://escs2014.iscbsc.org/escs-booklet.

## Keynotes

The consistent theme of the ESCS keynotes was the different aspects of dealing and interpreting the massive amounts of biological data that is nowadays available, often publicly and without restrictions of use. In the morning, Dr. Lennart Martens introduced the concept of 'Saprotrophics', a field that many bioinformaticians might be working in without realising it and that has even peaked the interest of the social sciences [2]. The central idea behind Saprotrophics is that, with the appropriate methods, new knowledge can be obtained from massive amounts of public data, in directions that go far beyond the original intention and purpose. Although such analyses come with their own set of unique challenges, these can be overcome with proper approaches. Dr. Martens gave an overview of such challenges, of possible ways to tackle them and in addition he showed some interesting applications.

The second keynote, by Dr. Jeroen de Ridder, underlined the critical importance of scale in biological data sets. Depending on the scale used to analyze and interpret data, the features and patterns that emerge can change quite dramatically; this is comparable to the change in perception we have of a landscape when we are flying over it or walking in it. Through an array of working examples Dr. de Ridder guided the audience into understanding that meaningful new insights in molecular data analyses can be achieved by accounting for the importance of scale and using scale-aware analyses tools.

Finally, the keynote from Dr. Lars Juhl Jensen concerned the efforts needed to collect and combine data from different sources into a single, biologically meaningful, network. In his talk, Dr. Jensen detailed the efforts and techniques that were necessary to construct the STRING database [3]. This database combines data derived from different curated databases, applying refined automatic text mining techniques and computational prediction approaches. Several of these methods have been integrated into web-based resources, which can be used to construct other databases and are extremely valuable for systems biology applications.

## Student presentations

From all abstracts submitted to the symposium, the best eight were selected for oral presentations, which were divided into three sessions.

### Session 1: Modeling

Information about the DNA replication mechanisms is scarce or absent for many viruses. Kazlauskas *et al*. [4] reported an analysis of DNA replication genes across more than 1500 viral genomes. This analysis allowed Kazlauskas and colleagues to identify previously unknown replication components in these genomes.

Conformation alterations are often a critical step for the functionality of a variety of proteins. Narunsky *et al*. [5] introduced ConTemplate, a web server able to suggest potential conformations for proteins with an established molecular structure based on structural similarity to other proteins with known conformations.

### Session 2: Systems biology

Proteochemometrics is the modelling of the bioactivity of ligands against different targets. Cortes *et al*. [6] demonstrated that a Bayesian inference scheme can be successfully applied to this problem within the contexts of isoform-selective cyclooxygenase inhibition and large-scale cancer cell line drug sensitivity.

Understanding the manner with which small compounds inhibit protein-protein interactions would greatly help in the design of the next generation of therapeutic compounds. Kuenemann *et al*. [7] studied small molecules and protein-protein interactions of such inhibitors to identify new putative 3D characteristics that support inhibition.

While rich information sources exist for protein interaction data, their adaptive nature remains poorly understood. Using advanced pattern mining techniques, Naulaerts *et al*. [8] discovered dynamic interaction patterns in lists of differentially expressed proteins that could be related to cancer states.

### Session 3: Networks and statistics

DNA methylation is an important epigenetic marker that has been shown to be involved in gene silencing. Döring *et al*. [9] modeled the differences in sequence bias that exist for methylation determination through microarray hybridization and bisulfite sequencing.

The identification of critical residues is of great interest for the field of protein engineering. Armenta-Medina *et al*. [10] introduced a hybrid approach called ANMA.SCA to determine the importance of a residue in proteins, based on coevolution and cross-correlation of simulated atomic fluctuations.

Gene duplications are notoriously hard to correctly position in phylogenetic reconstructions of the genomic evolutionary history. Peres *et al*. [11] have developed a new method to improve the positioning of gene duplication in gene trees produced by TreeBest.

## Award Winners

At ESCS, four awards were given to the best presenters of the day, namely two for oral presentations and two for poster presentations. The attendees determined the winners by scoring the different oral presentations based on presentation style, novelty of the work presented, slide layout and clarity of the message. The best presentation award went to Mélaine Kuenemann, while the runner-up prize went to Isidro Cortes. The best posters were selected during the noon poster session based on preferences expressed by the symposium attendants through stickers. The five top scoring posters were given the chance to give a 5 minutes flash presentation during the main meeting. From these five flash presentations, the award winners were determined by an independent jury. Poster presentation first place went to Jakob Jespersen, and second place to Aurélie Pirayre.

## Conclusions

As previous editions, the third ESCS was a great success, characterized by talks of high profile and quality, both at the level of keynotes and submitted work. This is confirmed by the results of an online survey that participants were asked to fill in. Most participants agree that the quality of the symposium was high to excellent, and that the equilibrium between keynotes and submitted talks was good. This year, we noted a decrease in the number of participants in comparison to ESCS of two years ago, similarly to what observed in this year's SCS [1]. An informal survey among students attending the main conference that didn't subscribe for ESCS showed that the main reasons for not attending were either conflicting workshops taking place on the same day or unfamiliarity with the Student Council and its activities. Considering this, we recommend the organizers of future symposia to implement sharp strategies to improve the dissemination of announcements concerning the event in order to reach a larger pool of potential delegates. We also observed that we received far more applications for the ESCS travel fellowships than we were able to provide. This, together with the explicit declaration in some of the applications that attending the symposium would only be possible upon travel fellowship awarding, suggests that the lack of funding contributed as well to the drop in the number of delegates and underlines the importance of maintaining and possibly expanding the Travel Fellowship program from ISCB and its SC. Overall, we received very positive responses from all attendees, with many comments on the high quality of the oral presentations, both from keynotes and students.

## Future perspectives

Next year the ISMB and ECCB conferences will be co-organised in Dublin, Ireland, in July 10th to 14th. This meeting will serve as the location for the 11^th ^SCS and therefore the next ESCS will only take place in 2016. For information on the Student Council and other events we organize for students in computational biology and bioinformatics, please visit our website: http://www.iscbsc.org.
